# Metabolite Genome-Wide Association in Hispanics with Obesity Reveals Genetic Risk and Interactions with Dietary Factors for Type 2 Diabetes

**DOI:** 10.3390/metabo15110697

**Published:** 2025-10-28

**Authors:** Chao-Qiang Lai, Laurence D. Parnell, Zhuoheng Li, Sabrina E. Noel, Shilpa N. Bhupathiraju, Katherine L. Tucker, José M. Ordovás

**Affiliations:** 1 USDA ARS, Nutrition and Genomics Laboratory, JM-US Department of Agriculture Human Nutrition Research Center on Aging at Tufts University, Boston, MA 02111, USA; laurence.parnell@usda.gov (L.D.P.); zl292@cornell.edu (Z.L.); jose.ordovas@tufts.edu (J.M.O.); 2 Department of Public Health and the Center for Population Health, University of Massachusetts Lowell, Lowell, MA 01854, USA; sabrina_noel@uml.edu (S.E.N.); katherine_tucker@uml.edu (K.L.T.); 3 Channing Division of Network Medicine, Brigham and Women’s Hospital and Harvard Medical School, Boston, MA 02115, USA; sbhupath@hsph.harvard.edu; 4 Department of Nutrition, Harvard T.H. Chan School of Public Health, Boston, MA 02115, USA; 5 IMDEA-Food Institute, CEI UAM+CSIC, 28049 Madrid, Spain

**Keywords:** metabolites, links between obesity and type 2 diabetes, gene–diet interaction, metabolite genome-wide association study

## Abstract

Background: Obesity is a leading cause of type 2 diabetes (T2D), with particularly high prevalence in Hispanic populations residing in the USA. However, how genetic variation influences obesity-related blood metabolite levels which, in turn, contribute to T2D progression, is not well understood. Our goal was to identify and understand genetic and dietary connections between obesity and T2D in a Hispanic cohort of older adults. Materials and Methods: We conducted a genome-wide association study on 13 specific metabolites previously associated with T2D and characteristic of individuals with abdominal obesity within the Boston Puerto Rican Health Study cohort. We further examined associations of identified metabolite quantitative trait loci (mQTLs) and their interactions with targeted dietary factors on T2D prevalence and related traits. We used gene set and pathway analysis with protein–protein interaction networks to explore the molecular mechanisms underlying the metabolic connections between obesity and T2D. Results: We identified 30 single-nucleotide polymorphisms (SNPs) acting as mQTLs for these 13 metabolites. These mQTLs were located within 19 gene regions, associated with processes such as linoleic acid metabolism, alpha-linolenic acid metabolism, and glycerophospholipid biosynthesis. Although no mQTLs were directly associated with T2D or related traits, 12 demonstrated interactions with certain food groups that affect T2D risk. Moreover, gene set and pathway analysis with protein–protein interaction networks indicated that alpha-linolenic acid metabolism, lipid metabolism, and glycerophospholipid biosynthesis and metabolism among other pathways are potential connections between T2D and obesity. Conclusions: This study identifies biochemical relationships between genetic susceptibility and dietary influences, contributing to our understanding of T2D progression in Hispanic people with obesity.

## 1. Introduction

Type 2 diabetes (T2D) remains a major public health concern, affecting millions of individuals globally [1]. One of its primary risk factors is obesity, which disrupts the metabolic and physiological mechanisms responsible for insulin responsiveness and glucose regulation [1,2,3]. Despite this recognized link, the detailed processes by which obesity induces insulin resistance remain elusive.

An analysis of T2D across various populations indicates a complex interplay of genetic, environmental, and cultural factors in its development and progression [4]. Notably, Hispanic, or Latino, populations in the U.S. experience significantly higher incidence of T2D compared to non-Hispanic White individuals [5]. A part of this heightened susceptibility can be attributed to a combination of genetic predispositions and specific lifestyle choices prevalent in the Hispanic communities [6,7]. However, a clear understanding of how dietary practices influence metabolic profiles and the subsequent risk of T2D in this demographic is still lacking. Addressing this knowledge gap is crucial for devising effective dietary strategies to mitigate T2D onset in this population.

Recent advances in metabolomics offer valuable insights into relationships between genetic factors, diet, and risk of T2D [8]. Using advanced techniques, numerous studies have identified metabolites associated with T2D risk, including branched-chain amino acids (BCAAs) and glycerolipids [9,10,11]. These metabolites reflect the intricate interactions that dictate health outcomes.

We previously performed metabolomic profiling in the Boston Puerto Rican Health Study (BPRHS) cohort to compare the risk of T2D between obese and non-obese participants [12]. We identified 13 metabolites that were uniquely associated with baseline T2D prevalence in participants with obesity, and with T2D incidence in the ~6-year follow-up. Each of these 13 metabolites correlated with at least one food group, such as sugar-sweetened beverages (SSB) and vegetable intake. Further research with the BPRHS and the San Juan Overweight Adult Longitudinal Study (SOALS) cohorts also revealed several metabolite clusters, including glucose transport, sphingolipids, acyl cholines, sugar metabolism, branched-chain and aromatic amino acids, and fatty acid biosynthesis, related to T2D prevalence in these Hispanic populations [13]. Building on these findings, the current study aims to uncover genetic determinants linking blood metabolites to T2D risk. We conducted a comprehensive genome-wide scan to identify relevant genetic variants or metabolite quantitative trait loci (mQTLs). Our goal was to investigate the genetic loci (mQTLs) that influence the levels of the 13 obesity-T2D metabolites, and to elucidate in a Hispanic population the associations of those loci with T2D risk, and any genotype by diet interaction that modifies that risk.

## 2. Study Population and Methods

Boston Puerto Rican Health Study (BPRHS): The flowchart of the study design is shown in Figure S1. The BPRHS is a longitudinal cohort study aimed at understanding the interplay between stress, nutrition, and health outcomes, such as metabolic and cardiovascular diseases, in Puerto Ricans residing in the greater Boston area [14]. From 2004 to 2015, interviews were conducted at three intervals: baseline, ~2 years post-enrollment, and ~6 years post-enrollment. Detailed methodologies on recruitment and data collection have been reported [14,15]. Comprehensive dietary, clinical, and biochemical assessments were conducted on 1303 out of 1504 adult participants, who were aged 45 to 75 years. Plasma samples of 806 participants were obtained for metabolomic profiling by Metabolon Inc. (Morrisville, NC, USA).

Dietary Assessment: Dietary intake was ascertained using a food frequency questionnaire (FFQ) tailored and validated for this Hispanic adult cohort [16]. The FFQ was adapted from the National Center Institute/Block FFQ format using dietary recall data for Puerto Rican adults from the Hispanic Health and Nutrition Examination Survey to add culturally specific foods and portion sizes. Mixed dishes were disaggregated into individual food groups, as performed previously. Daily average nutrient intakes from foods were calculated using the Nutrition Data System for Research Software (version 2016, Nutrition Coordinating Center, University of Minnesota). We organized 126 food items into 34 nutrient-based categories [15]. For instance, the “dairy desserts” category included dairy-based desserts such as cheesecake, ice cream, and puddings. Sugar-sweetened beverages (SSB) incorporated beverages with added sugar or artificial sweeteners, delineated into six sub-categories: (1) regular and caffeine-free cola; (2) sugary carbonated drinks; (3) fruit-flavored drinks, nectars, and punches, excluding 100% fruit juice; (4) vitamin-enriched drinks with added sugar; (5) pre-sweetened ready-to-drink tea; and (6) diet cola and non-cola drinks. Daily SSB intake was quantified in servings.

Obesity and type 2 diabetes assessment: Obesity was defined using waist circumference measurement, which has been shown to be a more reliable indicator of T2D risk than BMI [12,17]. Men and women with waist circumference ≥ 102 cm and ≥88 cm, respectively, were categorized as obese, whereas non-obese participants fell below these measurements.

Blood samples were collected after an overnight fast. Fasting glucose was measured using an enzymatic, kinetic reaction (OSCR6121; Olympus America, Melville, NY, USA). Glycosylated hemoglobin (HbA1c) was determined using a latex immunoagglutination inhibition method. Fasting serum insulin was assessed with an IMMULITE 1000 (Siemens Medical Solutions Diagnostics., Los Angeles, CA, USA). Type 2 diabetes (T2D) was defined as FPG ≥ 126 mg/dL, or self-reported use of hypoglycemic medications. HOMA-IR (homeostatic-model-assessment-insulin-resistance), as an insulin resistance index, was calculated according to the following formula: fasting insulin (microU/L) × fasting glucose (mg/dL)*0.055/22.5.

Metabolomic profiling: Plasma samples at baseline were collected from recruited BPRHS participants and stored at −80 °C. Metabolon Inc. performed metabolomic analysis on 806 plasma samples collected at baseline [12]. Briefly, after protein was extracted from the plasma, metabolomics analysis was performed using ultra-high-performance liquid chromatography–tandem mass spectrometry. Detection and identification of metabolites was performed by measuring the area under the peak curve against a library of more than 4500 purified standards containing retention time/index, mass-to-charge ratio, and chromatographic data. Measurements for each metabolite were normalized across all samples and validated by Metabolon Inc. [18].

Genotyping and genome-wide association study: GWAS genotyping in the BPRHS was conducted using the Affymetrix Axiom Genome-Wide LAT 1 Array, which was designed especially for Hispanic populations and contains probe sets to genotype 817,810 SNPs. GWAS genotypes were identified and QC was assessed using Genotype Console (GTC) and Affymetrix Power Tools (APT) in R [19] by following the standard protocol-Best Practices provided by the vendor. Based on the criteria of SNPolisher, 804,947 SNPs passed general QC. Among them, 712,197 were autosomal SNPs that met the following criteria: call rate ≥ 97%, minor allele frequency (MAF) ≥ 1%, *p*-value of Hardy–Weinberg equilibrium ≥ 10^−^^6^.

Principal components analysis (PCA) for population structure: To estimate the population structure, 50,704 SNPs were selected based on the following criteria: call rate > 97%, MAF ≥ 5%, pairwise linkage disequilibrium R squared ≤ 0.1, HWE *p* ≥ 10^−6^. Using principal components analysis implemented in SVS (GOLDENHELIX Inc. (Bozeman, MT, USA), the first principal component (PCA1) was selected to represent the population structure, based on the scree plot (eigenvalue ≥ 1). The PCA1 was included in all regression models to adjust the population structure.

Gene set and pathway analysis with protein–protein interaction networks: To complement pathways derived from the identified metabolites in the previous report [12], the 13 metabolites were assessed for pathway and functional group enrichment with the MBROLE platform [20]. Results from MBROLE included significantly enriched protein–metabolite interactions. We used these protein–metabolite interactions, filtered for those represented by three or more metabolites with an FDR-corrected *P* value, to build protein–protein interaction networks and discover the functional implications of this set of important metabolites, especially as most metabolic pathways are populated by metabolites and proteins [21]. Networks were built with the Human Reference Interactome (HuRI) tool [22] using default settings and the proteins from the significant protein–metabolite interactions. All input proteins, plus those protein interactions identified by Huri, underwent pathway analysis with Reactome [23] and g:Profiler tools [24] using default settings.

### Statistical Analysis

Genome-wide association of blood metabolite levels: To identify genetic variants (i.e., quantitative trait loci of metabolites, mQTLs) associated with the 13 obesity- and T2D-related metabolites (see Figure S1), we conducted a genome-wide association study with 712,197 SNPs that passed QC and met the required criteria (see GWAS genotyping): MAF > 0.01 in those participants (n = 560) who were classified as obese, based on waist circumference criteria. The GWAS for each of the 13 metabolites were conducted using mixed linear regression models with each metabolite as the outcome and SNP genotypes as predictors, with adjustment for sex, age, smoking, alcohol use, and population substructure. Each GWAS was implemented with the Golden Helix^®^ SNP & Variation Suite (SVS) 8.9.1. Multiple testing was corrected based on the Bonferroni test with genome-wide significance at *p* ≤ 5 × 10^−8^.

Association between mQTLs and type 2 diabetes and related traits: To determine if identified mQTLs are associated with T2D in the BPRHS (see Figure S1), we conducted an association study using logistic regression, with T2D as the outcome and mQTLs as the predictor, controlling for age, sex, smoking, alcohol use, physical activity, education, and population substructure in the participants with a full data set (n = 1300). For mQTLs with low minor allele frequencies (MAF < 0.05), a dominant model was applied whereby homozygotes and heterozygotes of the minor allele were combined into one group. Similarly, associations between mQTLs and related phenotypes were assessed in participants who did not use anti-diabetes medication, where T2D-related outcomes were log transformed and modeled as the outcome.

mQTLs by diet (GxD) interaction on type 2 diabetes and related traits: To examine if an mQTL exhibits an interaction with diet on T2D (see Figure S1), we conducted logistic or linear regression analysis, including interaction between identified mQTLs and food groups that were associated with metabolite levels, adjusting for age, sex, smoking, alcohol use, physical activity, education, and population substructure [12]. To increase the statistical power to detect GxD interactions, only mQTLs with MAF > 0.05 were examined and a dominant model was used. Multiple tests were corrected by the Bonferroni test.

To avoid the influence of anti-diabetes medication, we analyzed the mQTLs by diet interaction with T2D-related traits, such as fasting glucose, Hb1Ac, and HOMA-IR, only in participants who did not use such medication (n = 877). Moreover, because of the limited sample size, we restricted our analysis to mQTLs with MAF ≥ 0.05 for sufficient statistical power.

## 3. Results

### 3.1. Genome-Wide Association of 13 Metabolites

The T2D group had slightly higher age, obesity and hypertension prevalence, fasting glucose and HbA1c, and HOMA-IR than the non-T2D group (all *p* < 0.05) (Table 1). The T2D group also had slightly lower smoking and alcohol use. However, there were no significant differences in sex or total energy intake between the two groups, nor were significant differences observed in these characteristics between the metabolomic subsamples (n = 806) and the full cohort (n = 1303).

**Figure 1:**
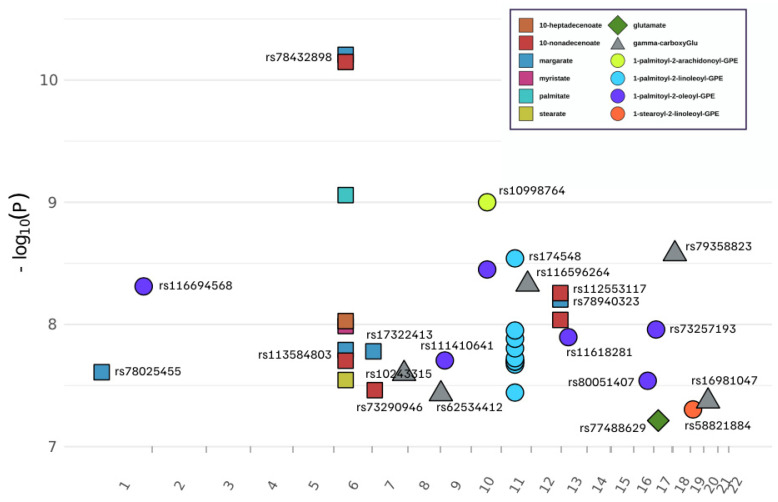
Genome-wide scan for metabolite quantitative trait loci (mQTLs) of 13 metabolites in BPRHS. Twelve of thirteen metabolites exhibited associations that passed the threshold of statistical significance at 38 loci. SNPs labeled with an rs identifier are those with regional peak association signals for the indicated metabolite. The *x*-axis indicates genome position by chromosome. SNP rs10998764 (Chr 10) had peak signals for both 1-palmitoy-2-arachidonoyl-GPE and 1-palmitoyl-2-oleoyl-GPE, and rs78432898 (chr 6) had peak signals for several fatty acids. For the plotted symbols, squares are for fatty acids, circles are for glycerophosphoethanolamines (GPEs), triangle represents gamma-carboxyglutamate, and the diamond is for the amino acid glutamate.

We performed a genome-wide association study (GWAS) to identify genetic variants (mQTLs) influencing the levels of each of 13 metabolites associated with T2D in individuals with obesity (n = 560). While controlling for sex, age, smoking, alcohol use, total energy, education, and population substructure, we found that plasma levels of 11 of 13 metabolites were significantly associated with at least one SNP at *p* ≤ 5 × 10^−8^ (Table 2, Figure 1). The GWAS QQ-plots of each metabolite (Figure S2A,B) strongly support the findings. The exception was 1-stearoyl-2-arachidonoyl-GPE (18:0/20:4). In total, we detected 38 associations of 30 distinct mQTLs with 12 metabolites that passed genome-wide significance (Table 2 and Figure 1).

For two glutamate metabolites (Table 2, glutamate and gamma-carboxyglutamate), five independent SNPs (mQTLs) in five genic regions (*TAS2R40*, *KIAA0020*, *SCN2B*, *LOC105371988*, *SLC24A3*), were associated with gamma-carboxyglutamate, a product of post-translational modification. A single SNP rs77488629 at *SLC47A1* was associated with glutamate with *p *= 6.12 × 10^−8^, which almost reached genome-wide significance.

For the six metabolites that can be grouped as long-chain fatty acids, seven mQTLs, represented by five genic regions (*ID3*, *TRAM2*, *ZNF815P*, *TMEM132C*, *TMEM106B*), were identified to be associated with these metabolites: margarate (17:0), myristate (14:0), palmitate (16:0), stearate (18:0), 10-heptadecenoate (17:1n7), and 10-nonadecenoate (19:1n9) (Table 2).

Four metabolites in the phosphatidylethanolamine (PE) synthesis pathway yielded 18 mQTLs in 11 genic regions (*TACR2*, *MYRF*, *TMEM258*, *FADS1*, *FADS2*, *TRAF5*, *MLLT3*, *TACR2*, *MEDAG*, *GLP2R*, *SMARCA4*). One metabolite, 1-palmitoyl-2-linoleoyl-GPE (16:0/18:2), was consistently associated with 10 SNPs in four genes (*MYRF*, *TMEM258*, *FADS1*, *FADS2*), all very near neighbors mapping to the q12.2 region of chromosome 11. In each case, MAF was ≥ 0.369 and all *p* < 3.6 × 10^−8^. Six SNPs in the *FADS1* region were in strong linkage disequilibrium, representing one common variant in the *FADS1* gene, rs174548. In addition, 1-palmitoyl-2-oleoyl-GPE (16:0/18:1) was significantly associated with six mQTLs located in six genic regions (*TRAF5*, *MLLT3*, *TACR2*, *MEDAG*, *GLP2R*, and *CDH8*).

### 3.2. Associations Between mQTLs and T2D Risk

To determine if the identified 30 mQTLs contribute to T2D prevalence in the BPRHS, we conducted a new association study using a logistic regression model, with T2D as the outcome and 30 mQTLs as predictors, controlling for potential confounding factors in the full sample (n = 1300). None of the 30 mQTLs showed significant association with T2D (*p* > 0.05). Similarly, none of 30 mQTLs were associated with fasting glucose, HOMA-IR, or HbA1c in participants who did not use anti-diabetes medication, controlling for potential cofounding factors (*p* > 0.05, n = 877).

### 3.3. Gene by Diet (GxD) Interaction on T2D Between mQTLs and Dietary Factors Associated with Obesity Metabolites

Genetic risk of T2D often depends on environmental factors, and dietary habits constitute one group of those factors [25]. Thus, to understand the relationship between dietary intake and risk of T2D, we tested for interactions between mQTLs and those food groups that were associated with the 13 obesity-related metabolites to determine the risk of T2D [12]. For dietary intake, we focused on the 13 food groups that were associated with 13 metabolites, as reported [12]. Adjusting for potential cofounding factors (sex, age, smoking, alcohol use, physical activity, total energy intake, education, and population substructure) and using linear regression models, we found that 22 of 30 mQTLs, representing 13 genes, showed nominally significant interaction at *p* < 0.01 with 12 food groups (Table S1). The strongest of the detected interactions involved *SLC47A1* variant rs77488629 and intake of dairy desserts, with *p* = 3.63 × 10^−4^. Notably, 11 SNPs, representing at least five genes (*MLLT3*, *MYRF*, *TMEM258*, *FADS1*, *TMEM132C*), exhibited consistent interactions (on increased risk) with SSB intake on T2D.

**Figure 2:**
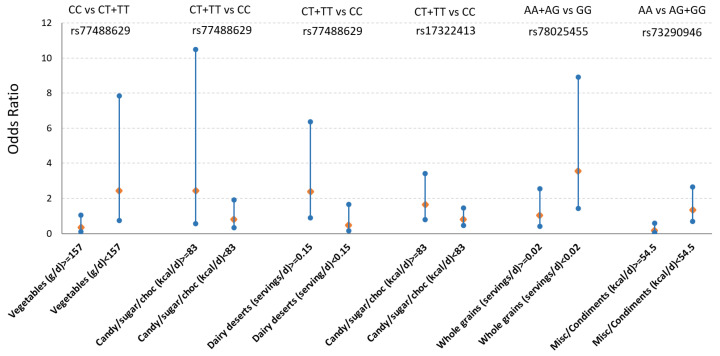
Interaction between mQTLs and food groups on type 2 diabetes. Odds ratios were calculated for the comparison of two genotype groups (i.e., dominant model) using a logistic regression model when applying the threshold of food intake mean of the population, while adjusting for potential confounders (sex, age, alcohol use, smoking, physical activity, total energy intake, education, and population structure). Orange diamonds indicate odds ratios and blue lines indicate the lower and upper 95% ranges. These six interactions between the four mQTLs (*SLC47A1*-rs77488629, *ZNF815P*-rs17322413, *ID3*-rs78025455, *TMEM106B*-rs73290946) and five food groups were significant after multiple testing correction with a *p* value < 0.002 (Table S1).

As the 30 mQTLs were not completely independent, we determined that these mQTLs represented 22 independent mQTLs, based on the correlation matrix of pairwise linkage disequilibrium among the 30 mQTLs [26]. Hence, after correction for multiple testing (*p* = 0.05/22, ≤ 0.0023), four mQTLs displayed significant interaction with five distinct food groups on T2D (Figure 2 and Table S1). For the T carriers (CT + TT) of *SLC47A1*-rs77488629 showed increased risk of T2D when consuming more sweets (≥83 kcal/d, odds ratio (OR) = 2.44 vs. 0.81) or dairy desserts (≥0.15 servings/d, OR = 2.37 vs. 0.49), compared to CC carriers. In contrast, CC carriers had lower T2D risk when consuming more vegetables than T-carriers. Similarly, T-allele carriers (CT + TT) of rs17322413 had greater risk of T2D when consuming more sweets (≥ 83 kcal/d, OR = 1.65 vs. 0.81). Lastly, A allele carriers (AA + AG) of *ID3*-rs78025455 had greater risk of T2D when eating less than 0.02 servings/d of whole grain foods, compared to GG carriers.

**Figure 3:**
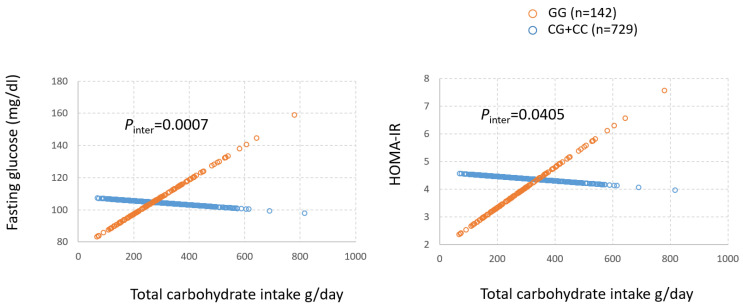
Interaction between *FADS1*-rs174548 and total carbohydrate intake on fasting glucose and insulin resistance index (HOMA-IR). Fasting plasma glucose concentrations and HOMA-IR were estimated by the two genotypes of *FADS1*-rs174548 (orange circles = GG and blue circles = CG + CC) based on regression models while adjusting for potential confounders (sex, age, alcohol use, smoking, physical activity, total energy intake, education, and population substructure). The predicted values (*y*-axis) were then plotted against total carbohydrate intake (grams/day, *x*-axis).

T2D risk is assessed by a variety of clinical measures. Thus, we performed GxD analysis to investigate if mQTLs by diet interaction were associated with T2D-related outcomes, such as fasting glucose, Hb1Ac, and insulin resistance index (HOMA-IR). We excluded participants who used anti-diabetes medication and focused on mQTLs with MAF ≥ 0.05. The only mQTLs that satisfied this condition were in the FADS regions (Table S2). For fasting glucose, all mQTLs at the *FADS1*/*FADS2* region showed strong GxD interaction with SSB intake (Table S2) with *p* ≤ 1.09 × 10^−7^, controlling for potential confounding factors (age, sex, smoking, alcohol use, education, physical activity, total energy intake, and population structure). For the insulin resistance index HOMA-IR, these mQTLs displayed similarly strong interactions (*p* ≤ 6.27 × 10^−5^). However, there were no significant interaction effects on Hb1Ac. As total carbohydrate intake was highly correlated with SSB intake (r = 0.79), we further characterized these mQTLs at *FADS1* as exhibiting similar interactions with total carbohydrate intake on fasting glucose and HOMA-IR (Table S1 and Figure 3). For the representative mQTL at *FADS1*, rs174548 (Table 2 and Table S1), the GG carriers exhibited greater risk of T2D with greater consumption of total carbohydrate, whereas the C-allele carriers (GC + CC) did not show the same trend (Figure 3).

A question naturally arises as to whether the identified SNPs are functional in some way that suggests an allele-specific response to the identified foods. To explore this, we queried the 6 mQTLs and the 11 SSB SNPs at the GTEx Genotype-Tissue Expression resource to identify any tissue-specific, allele-based expression of genes (eQTL). SNP rs17322413 associated moderately with expression of *SNORA42* in brain, and *PMS2* in tibial nerve, tibial artery, and skin. Regarding the SSB variants, across the *FADS1*/*FADS2* region, there are many very strong eQTLs, including rs174549, with its strongest signal for *FADS2* expression in whole blood and interaction with SSB intake, and rs174555 with the strongest association with *FADS1* expression in pancreas.

### 3.4. Pathways Represented by Metabolite–Protein Networks

**Figure 4:**
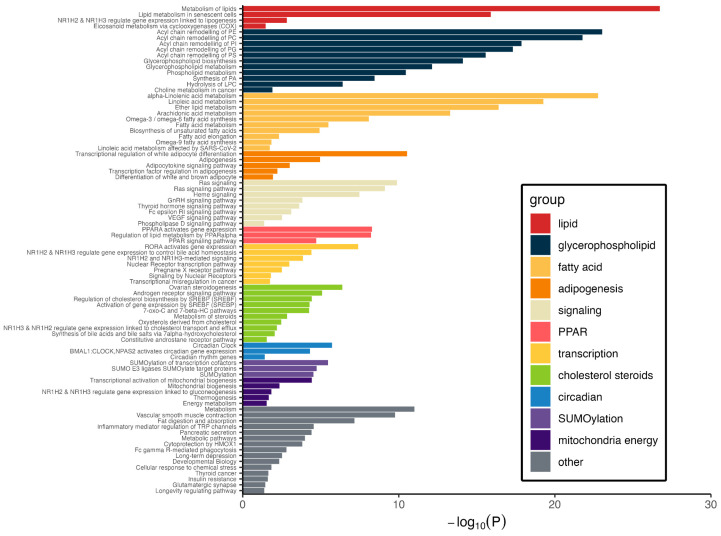
Pathways and bioprocesses represented by and significantly enriched in a metabolite-derived protein–protein interaction network. Proteins identified as interacting with three or more of the 13 obesity-related, T2D-relevant metabolites served as input to identify protein interactors. The set of proteins interacting with metabolites, the protein–protein interactors, and the proteins encoded by the genes identified by mQTL analysis were (n = 260) subjected to pathway enrichment analysis (see main text for details). Significantly enriched pathways are organized functionally by metabolism or bioprocess group. A corrected *p* value < 0.05 was considered significant.

We previously identified these 13 metabolites and described their functions based solely on the metabolites themselves. Here, we expand that functional assessment by including proteins that interact with the metabolites and using those proteins as seeds to build a protein–protein interaction (PPI) network suitable for analysis, for which pathways and bioprocesses were represented therein [21]. MBROLE identified 60 proteins that passed filters for interacting with three or more metabolites per protein interaction and FDR for statistical significance. The HuRI resource was used to identify PPIs. Only 35 of the 60 input proteins had PPI data in HuRI, and they produced 838 PPI pairs and 212 unique proteins. To assess pathway representation by this metabolite-inspired PPI network, these 212 proteins were complemented with the 25 proteins without PPI data and the 23 proteins encoded by genes identified in the mQTL GWAS (Table 2). Several pathways and bioprocesses were significantly represented in the PPI network (Figure 4 and Table S3). These included glycerophospholipid biosynthesis (7.8 × 10^−15^), and particularly its offspring in terms of acyl chain remodeling of phosphatidylethanolamine (9.3 × 10^−24^) and phosphatidylcholine (1.7 × 10^−22^), alpha-linolenic acid metabolism (1.7 × 10^−23^), PPARA activates gene expression (5.2 × 10^−9^), SUMOylation of transcription cofactors (3.5 × 10^−6^), and the circadian clock (1.9 × 10^−6^). This network is also significantly enriched for rate-limited enzymes, including FADS2 and 16 others [27], whose enzymatic properties may indicate that pathway flux is acutely sensitive to constituent metabolite levels. Furthermore, although three pathways for NRF2 (also known as NFE2L2), namely the NRF2 pathway, nuclear events mediated by NFE2L2 and the KEAP1-NFE2L2 pathway were not observed as significantly represented by the PPI network (*p* > 0.99), and the single significantly enriched transcription factor binding motif for the genes encoding PPI elements was for NRF2 (*p* corrected = 0.028). This motif was identified in 31 of 260 genes. Overall, these results highlight hidden bioprocesses and activities that cannot be identified by metabolites alone, but by a moderately sized protein network based on protein–metabolite interactions.

## 4. Discussion

Understanding the high prevalence of T2D in Hispanic populations with obesity requires scrutiny of genetic factors, diet, and blood metabolites. We undertook a GWAS on 13 metabolites we previously had linked with T2D and found those uniquely associated with abdominal obesity [12]. These metabolites revolved around three classes: glutamate, long-chain fatty acid metabolism, and phosphatidylethanolamine metabolism. We identified 30 mQTLs spanning 19 genic regions for these metabolites. While no direct T2D association was found with these mQTLs, 12 showed interactions with food groups linked to metabolites affecting T2D risk, implicating genes in pathways like alpha-linolenic acid metabolism, lipid metabolism, glycerophospholipid biosynthesis and metabolism. Essentially, our results highlight the metabolic bridges between genetic risk, dietary factors, and T2D in Hispanic people with obesity.

Earlier GWAS identified genetic risk factors for T2D primarily in non-Hispanic White populations, but many of these discoveries have not been observed in Hispanic groups [28,29]. Factors like ancestry, traditions, diet, and gene–diet interactions could explain this disparity [28,30]. Our objective was to describe mechanisms behind the high T2D prevalence in Hispanic populations. Abdominal obesity is a major risk factor for T2D in this population [12], and we established a connection between 13 metabolites and T2D in individuals with central obesity. The observed gene–diet interactions have more influence on T2D risk than the genes themselves. This suggests that there are food groups that either mitigate or exacerbate T2D risk for this demographic, and often in an allele-specific manner.

Notably, none of identified 30 mQTLs related directly to heightened T2D risk in this Hispanic population. However, 22 did show nominal or significant interactions with dietary food groups on risk of T2D, stressing the role of diet in T2D risk. For example, SSB intake has been identified as an obesity factor [31,32] in this population via phosphatidylcholine and lysophospholipid pathways, thereby linking SSB intake to obesity (Zhou et al., 2020) [33]. The over-represented pathways exemplified by the 13 T2D-associated metabolites unique in the participants with obesity are mapped to the phosphatidylcholine (PE) metabolic pathway (Parnell et al., 2021) [12]. This study revealed that four metabolites in the PE pathway are associated with 11 genetic loci (Table 2). Among them, eight loci are mapped to *FADS1* and *FADS2* gene regions and are in strong LD. These observations illustrate links between diet and T2D risk through genetic factors and the metabolomic network.

Obesity’s promotion of T2D largely stems from insulin resistance, a complex interplay that is not yet fully understood [3]. In this study, the mQTLs at *FADS1*, each with high minor allele frequency, between 0.33 and 0.42, displayed GxD interaction with SSB intake and total carbohydrate intake on T2D risk, and T2D-related phenotypes, i.e., glucose and HOMA-IR, in participants who were not using anti-diabetes medication. Homozygotes for the minor allele (GG) showed elevated fasting glucose and HOMA-IR when either SSB intake or total carbohydrate intake increased (Figure 3), whereas carriers of the major allele did not. This supports a central function for FADS activity in controlling insulin resistance. We noted that *FADS1* interacts with dietary intake, such as SSB and carbohydrate intake, possibly leading to insulin resistance, and others have proposed polyunsaturated fatty acids and fatty acid desaturase (FADS) activity [34].

To extend the analysis of metabolites and the bioprocesses in which they function, providing insight into the mechanisms by which obesity leads to T2D, we built a modest protein–protein interaction network seeded by proteins that have a physical association with 3 or more of the 13 metabolites, as described above. This network of 260 proteins is enriched in several biological pathways and processes, including glycerophospholipid biosynthesis and, in particular, acyl chain remodeling of phosphatidylethanolamine, alpha-linolenic acid metabolism, gene expression regulated by PPARA, SUMOylation of transcription cofactors, and circadian clock. It has been reported that treatment of primary rat adipocytes with insulin had sharp effects on the activity of mitochondrial GPAM, which catalyzes a rate-limiting step in triacylglycerol and glycerophospholipid biosynthesis by increasing V_max_ and K_m_ for the substrates glycerol-3-phosphate and palmitoyl-CoA [35]. A consequence of an elevated Km is reduced affinity of the enzyme for its substrate, which would affect the production of PE, PC and other 1,2-diacylglycerophospholipids.

The transcriptional regulator PPARA, or peroxisome proliferator-activated receptor alpha, participates broadly in several of the bioprocesses represented by our results. For instance, PPARA is a direct regulator of several core clock components, thereby linking circadian rhythms to metabolism [36], which supports the finding of several PPARA variants associating with reduced risk of T2D in an elderly Greek population [37]. In addition, it has been reported that a PPARA response element exists within an evolutionarily conserved region of the *PEMT* promoter, a gene encoding the enzyme responsible for the conversion of phosphatidylethanolamine to phosphatidylcholine [38]. We demonstrated that carriers of the rs72828480 T-allele in *PEMT* had increased BMI with increased intake of sugar-sweetened beverages [33]. In skeletal muscle and adipose, induction of insulin secretion and action, as initiated by alpha-linolenic acid, depends on PPARA action in the liver [39]. The deSUMOylation of PPARA was shown to promote ubiquitin-mediated degradation of PPARA which, in turn, inhibited *FGF21* expression and fatty acid oxidation [40]. SUMOylation is marked by the covalent attachment of a small ubiquitin-like modifier (SUMO) peptide to a protein substrate via a lysine residue [41]. Studies have demonstrated the importance of SUMOylation in the maintenance of pancreatic beta cell functions by regulating transcription [42], oxidative stress [43], and insulin granule exocytosis [44,45]. Phosphorylation of mitogen-activated protein kinase 7 (MAPK7, or ERK5) induces SUMOylation of NFE2L2, which inhibits activity of the latter [46], while sumoylation of TCF7L2 enhances the interaction between TCF7L2 and beta-catenin [47]. Lastly, in addition to PPARA connections to the circadian clock described above, several studies by us and others have identified the involvement of variants in clock genes associated with obesity, weight loss and insulin resistance or T2D [48,49,50]. To summarize, our observations of the involvement of various pathways and bioprocesses in obesity-T2D relationships fit well with previous reports. Here, we extend that knowledge to include dietary factors that significantly correlate with these blood metabolites.

Further analysis of the metabolite–protein–protein interaction network revealed modest enrichment for genes with binding sites for transcriptional regulator NFE2L2. Nearly one in eight genes (31 of 260) in the network contain an NFE2L2 recognition motif. The evidence for involvement of NFE2L2 in metabolic processes relevant to obesity and T2D is vast, with primary functions in protecting against oxidative stress and electrophilic stress, and regulating lipid metabolism and cell inflammatory responses [51]. In addition, this transcription factor has been shown to protect mitochondria from oxidative stress-induced decay [52], which would have effects on numerous metabolic processes, including fatty acid oxidation. Treatment of Hek-293 kidney cells with flavonoids quercetin, kaempferol, fisetin, daidzein, luteolin and apigenin at 20 μM concentration was observed to increase *NFE2L2* promoter activity significantly [53]. Fruits, vegetables, tea, and wine are primary sources of flavonoids in most diets and cultures. *TCF7L2* encodes a transcription factor that is important in Wnt signaling, and its genetic variants have been associated with risk of T2D in humans [54]. Genome-wide distribution of TCF7L2 binding and gene expression analysis in adipocytes point to direct regulation by TCF7L2 of genes implicated in cellular metabolism and cell cycle control, including NFE2L2. When challenged with a high-fat diet, conditional deletion of *TCF7L2* in adipocytes led to impaired glucose tolerance, impaired insulin sensitivity, weight gain, and increased adipose tissue mass [55].

Early efforts to link blood metabolomics to cardiovascular disease risk were performed in the Kooperative Gesundheitsforschung in the der Region Augsburg (KORA) study, and one of those investigations identified robust correlations between metabolite levels and gene expression [56]. Considering KORA expression data correlating with two or more of the metabolites investigated here, we noted that the genes encode proteins functioning in mitochondrial long-chain fatty acid beta-oxidation and carnitine metabolism, and are most relevant to the hypoketotic hypoglycemia disorder [24].

## 5. Limitations

Our study is not without limitations. We relied on overnight fasting blood samples for targeted metabolomics, potentially missing information from other sample types or post-meal metabolomics. Targeted metabolomics is itself inherently limiting in terms of the metabolites that are evaluated. The sample size of our study, from the BPRHS, may also have constrained the power of our analysis. BPRHS does not have the data for 2 h oral glucose tolerance test which could refine these results. Nonetheless, the simultaneous capture of metabolomic, genomic, and dietary data within one timeframe is an advantage, and more so in a population with distinct health disparities [57]. None of the identified 30 mQTLs related directly to increased T2D risk in this population, which should be considered when interpreting findings but also highlights the importance of diet for T2D risk. Finally, the findings might be specific to this population, potentially limiting generalizability. This warrants application of this approach and further validation in diverse population groups, including other Hispanic and non-Hispanic groups.

## 6. Conclusions

We integrated metabolomic and genomic data with dietary information to elucidate associations between genetic and dietary factors and increased risk of T2D in Hispanic people with obesity. We identified 22 mQTLs in 19 gene regions associated with aspects of dietary intake with T2D risk through 13 metabolites, highlighting a significant aspect of lipid metabolism bioprocesses.

## Figures and Tables

**Table 1:** Characteristics of participants in the BPRHS, by type 2 diabetes status and by metabolomic analysis.

		Type 2 Diabetes (T2D, n = 520)	No Diabetes (Non-T2D, n = 783)	Metabolomics (n = 806)	Full Cohort (n = 1303)
Age (SD)		58.8 (7.2) *	56.2 (7.7)	57.2 (7.4)	57.2 (7.6)
Female (%)		365 (70.2%)	563 (71.9%)	570 (70.7%)	928 (71.2%)
Body mass index (BMI, SD)		33.6 (6.8) *	30.9 (6.2)	32.1 (6.7)	31.9 (6.6)
Waist (cm)		106.3 (15.0) *	98.9 (13.9)	102.2 (14.8)	101.8 (14.8)
Obese ^#^ (n, %)		414 (79.6%) *	519 (66.2%)	584 (72.5%)	933 (71.6%)
Fasting glucose (mg/dL)		155.9 (69.6) *	97.1 (11.2)	119.9 (49.9)	120.4 (53.2)
Fasting insulin (mg/dL)		24.0 (35.7) *	14.1 (9.3)	18.5 (24.6)	18.0 (24.2)
Glycosylated hemoglobin (HbA1c, %)		8.3 (2.0) *	6.1 (0.8)	7.0 (1.7)	7.0 (1.8)
HOMA-IR		9.9 (24.3) *	3.4 (2.5)	5.9 (9.3)	6.0 (15.8)
T2D medication		431 (82.9%) *	0	254 (31.5%)	431 (33.1%)
Hypertension (n, %)		439 (84.4%) *	459 (58.6%)	448 (52.1%)	719 (55.2%)
Smoking (n, %)	Non-smoker	241 (46.3%)	347 (44.3%)	373	588
	Past smoker	167 (32.1%)	231 (29.5%)	248	398
	Current smoker	112 (21.5%) *	203 (25.9%)	183	315
Alcohol use (n, %)	Non-drinker	161 (31.0%)	219 (28.0%)	226	380
	Past-drinker	185 (35.6%)	209 (26.7%)	243	394
	Current drinker	172 (33.1%) *	351 (44.8%)	334	523
Education		2.4 (1.0) *	2.6 (1.0)	2.5 (1.0)	2.5 (1.0)
Physical activity score		30.8 (4.3) *	31.8 (4.9)	31.5 (4.7)	31.4 (4.7)
Total energy intake (kcal, SD)		2076 (887)	2153 (897)	2174 (975)	2122 (894)

^#^ The number of participants with obesity based on waist circumference ≥ 102 cm for men or ≥88 cm for women. * Significant difference at *t*-test (*p* < 0.05) between the non-T2D and type 2 diabetes groups.

**Table 2:** 30 Metabolite QTLs (mQTLs) of 13 metabolites from GWAS in participants (n = 560) with obesity.

Metabolite Dass	COmpID	Metabolite	SNP	Chr	Position *	Associated Gene	*p*-Value	SNP Beta	SNP Beta SE	Minor/Major	MAF ^#^
Glutamate Metabolism	57	glutamate	rs77488629	17	19448308	SLC47Al	6.12 × 10^−8^	0.164	0.030	T/C	0.015
	38754	gamma-carboxyglutamate	rs10243315	7	142900460	TAS2R40	2.53 × 10^−8^	0.621	0.110	C/T	0.012
	38754	gamma-carboxyglutamate	rs62534412	9	2869593	KIAA0020	3.71 × 10^−8^	0.634	0.114	C/T	0.011
	38754	gamma-carboxyglutamate	rs116596264	11	118043675	SCN2B	4.70 × 10^−9^	0.623	0.105	C/T	0.013
	38754	gamma-carboxyglutamate	rs79358823	18	10407315	LOC105371988	2.65 × 10^−9^	0.652	0.108	C/T	0.010
	38754	gamma-carboxyglutamate	rs16981047	20	19708935	SLC24A3	4.25 × 10^−8^	0.512	0.092	C/G	0.014
Long Chain Fatty Acid	1121	margarate (17:0)	rs78025455	1	23890507	ID3	2.46 × 10^−8^	0.458	0.081	A/G	0.014
	1121	margarate (17:0)	rs113584803	6	52435180	TRAM2	1.62 × 10^−8^	0.407	0.071	G/A	0.019
	1121	margarate (17:0)	rs78432898	6	52574289	?	6.22 × 10^−11^	0.441	0.066	C/T	0.021
	1121	margarate (17:0)	rs17322413	7	5879004	ZNF815P	1.66 × 10^−8^	0.334	0.058	T/C	0.029
	1121	margarate (17:0)	rs78940323	12	129114888	TMEM132C	6.25 × 10^−9^	0.407	0.069	A/G	0.018
	1365	myristate (14:0)	rs78432898	6	52574289	?	1.03 × 10^−8^	0.484	0.083	C/T	0.021
	1336	palmitate (16:0)	rs78432898	6	52574289	?	8.74 × 10^−10^	0.342	0.055	C/T	0.021
	1358	stearate (18:0)	rs78432898	6	52574289	?	2.85 × 10^−8^	0.203	0.036	C/T	0.021
	33971	10-heptadecenoate (17:1n7)	rs78432898	6	52574289	?	9.38 × 10^−9^	0.641	0.110	C/T	0.021
	33972	10-nonadecenoate (19:1n9)	rs113584803	6	52435180	TRAM2	1.98 × 10^−8^	0.625	0.110	G/A	0.019
	33972	10-nonadecenoate (19:1n9)	rs78432898	6	52574289	?	7.11 × 10^−11^	0.679	0.102	C/T	0.021
	33972	10-nonadecenoate (19:1n9)	rs73290946	7	12023844	TMEM106B	3.45 × 10^−8^	0.541	0.097	G/A	0.022
	33972	10-nonadecenoate (19:1n9)	rs78940323	12	129114888	TMEM132C	9.18 × 10^−9^	0.622	0.107	A/G	0.018
	33972	10-nonadecenoate (19:1n9)	rs112553117	12	129117140	TMEM132C	5.54 × 10^−9^	0.555	0.094	T/C	0.024
Phosphatidyle thanolamine (PE)	52464	1-palmitoyl-2-arachidonoyl-GPE (16:0/20:4)	rs10998764	10	71170572	TACR2	1.00 × 10^−9^	1.332	0.214	A/G	0.010
	42449	1-palmitoyl-2-linoleoyl-GPE (16:0/18:2)	rs174533	11	61549025	MYRF	2.13 × 10^−8^	0.388	0.068	A/G	0.341
	42449	1-palmitoyl-2-linoleoyl-GPE (16:0/18:2)	rs102274	11	61557826	TMEM258	1.12 × 10^−8^	0.398	0.069	C/T	0.341
	42449	1-palmitoyl-2-linoleoyl-GPE (16:0/18:2)	rs174546	11	61569830	FADS1	2.01 × 10^−8^	0.390	0.068	T/C	0.341
	42449	1-palmitoyl-2-linoleoyl-GPE (16:0/18:2)	rs174547	11	61570783	FADS1	2.01 × 10^−8^	0.390	0.068	C/T	0.342
	42449	1-palmitoyl-2-linoleoyl-GPE (16:0/18:2)	rs174548	11	61571348	FADS1	2.88 × 10^−9^	0.402	0.067	G/C	0.403
	42449	1-palmitoyl-2-linoleoyl-GPE (16:0/18:2)	rs174549	11	61571382	FADS1	1.57 × 10^−8^	0.396	0.069	A/G	0.333
	42449	1-palmitoyl-2-linoleoyl-GPE (16:0/18:2)	rs174550	11	61571478	FADS1	2.01 × 10^−8^	0.390	0.068	C/T	0.341
	42449	1-palmitoyl-2-linoleoyl-GPE (16:0/18:2)	rs174555	11	61579760	FADS1	1.89 × 10^−8^	0.392	0.069	C/T	0.335
	42449	1-palmitoyl-2-linoleoyl-GPE (16:0/18:2)	rs174566	11	61592362	FADS2	3.61 × 10^−8^	0.369	0.066	G/A	0.421
	42449	1-palmitoyl-2-linoleoyl-GPE (16:0/18:2)	rs174570	11	61597212	FADS2	1.31 × 10^−8^	0.429	0.074	T/C	0.239
	19263	1-palmitoyl-2-oleoyl-GPE (16:0/18:1)	rs116694568	1	211536212	TRAF5	4.88 × 10^−9^	1.765	0.297	G/A	0.016
	19263	1-palmitoyl-2-oleoyl-GPE (16:0/18:1)	rs111410641	9	20368408	MLLT3	1.97 × 10^−8^	0.837	0.147	T/C	0.061
	19263	1-palmitoyl-2-oleoyl-GPE (16:0/18:1)	rs10998764	10	71170572	TACR2	3.55 × 10^−9^	2.080	0.347	A/G	0.010
	19263	1-palmitoyl-2-oleoyl-GPE (16:0/18:1)	rs11618281	13	31488958	MEDAG	1.27 × 10^−8^	1.373	0.238	A/G	0.026
	19263	1-palmitoyl-2-oleoyl-GPE (16:0/18:1)	rs80051407	16	62592922	?	2.89 × 10^−8^	1.890	0.336	G/T	0.013
	19263	1-palmitoyl-2-oleoyl-GPE (16:0/18:1)	rs73257193	17	9779676	GLP2R	1.10 × 10^−8^	2.045	0.353	A/G	0.012
	52446	1-stearoyl-2-linoleoyl-GPE (18:0/18:2)	rs58821884	19	11159230	SMARCA4	4.96 × 10^−8^	1.065	0.193	A/G	0.021

* Position was based on Genome Built 37. ^#^ MAF = Minor allele frequeancy.

## Data Availability

The data used in this study are available upon reasonable request and review due to privacy and ethical considerations.

## Supplementary Materials

The following supporting information can be downloaded at https://www.mdpi.com/article/10.3390/metabo15110697/s1, Figure S1: Flowchart of study design. Supplemental Figure S2A,B: QQ plots of genome-wide association study of 12 metabolites. Supplemental Table S1. Interaction between mQTLs and food intake on type 2 diabetes (n = 1300). Supplemental Table S2. Interaction between mQTLs and food intake and diabetes-related traits in participants not taking anti-diabetes medication (n = 877). Supplemental Table S3: Pathways significantly enriched in the metabolite-derived protein–protein interaction network.

## Abbreviations

BMI	Body mass index.
BPRHS	Boston Puerto Rican Health Study.
Chr	Chromosome.
eQTL	Quantitative trait loci for allele-based expression of genes.
FFQ	Food frequency questionnaire.
FPG	Fasting plasma glucose.
GWAS	Genome-wide association study.
GxD	Gene by diet interaction.
GTEx	Genotype-Tissue Expression resource.
HbA1c	Glycosylated hemoglobin.
HOMA-IR	Homeostatic-model-assessment-insulin-resistance.
MAF	Minor allele frequency.
mQTLs	Metabolite quantitative trait loci.
OR	Odds ratio.
PC	Phosphatidylcholine.
PCA	Principal components analysis.
PE	Phosphatidylethanolamine.
PPARA	Peroxisome proliferator-activated receptor alpha.
PPI	Protein–protein interaction network.
SSB	Sugar-sweetened beverage.
SNP	Single-nucleotide polymorphism.
SSB	Sugar-sweetened beverages.
T2D	Type 2 diabetes.
