# Supplementary material for: Metabolite Genome-Wide Association in Hispanics with Obesity Reveals Genetic Risk and Interactions with Dietary Factors for Type 2 Diabetes
Source: Metabolites. 2025 Oct 28;15(11):697. doi: 10.3390/metabo15110697 (PMC12654851; doi:10.3390/metabo15110697)

Supplemental Figure 1: Flowchart of the study design

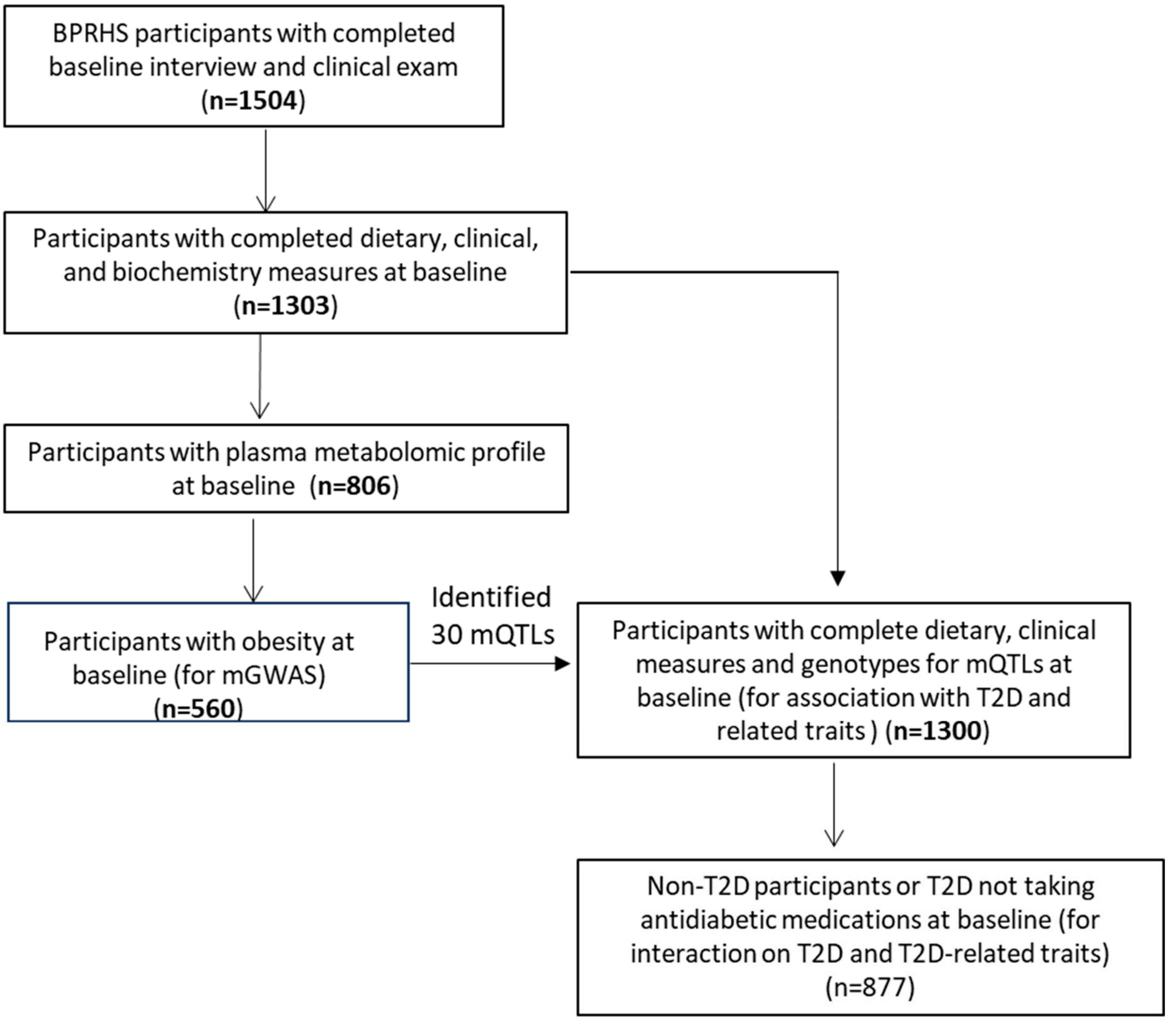

Supplemental Figure 2A:
QQ plots of genome-wide association study of metabolites

glutamate

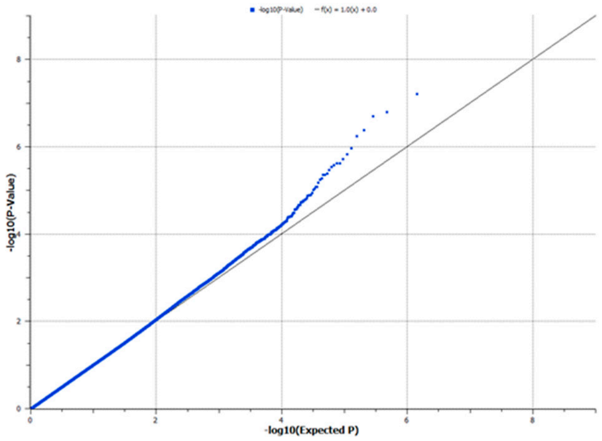

gamma-carboxyglutamate

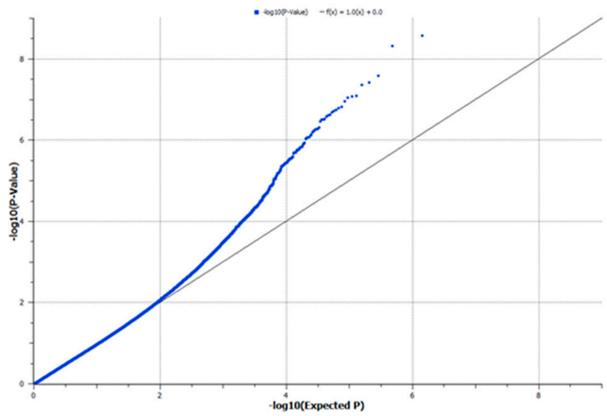

margarate

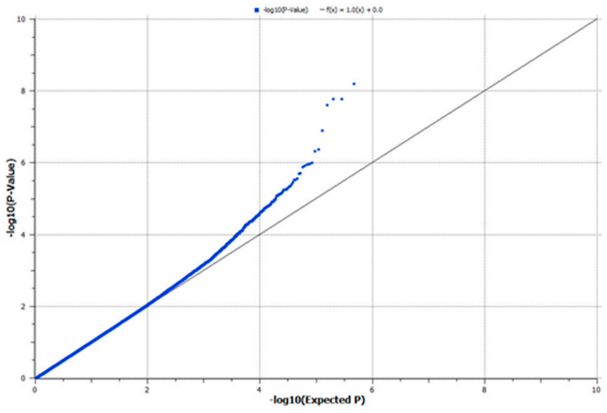

myristate

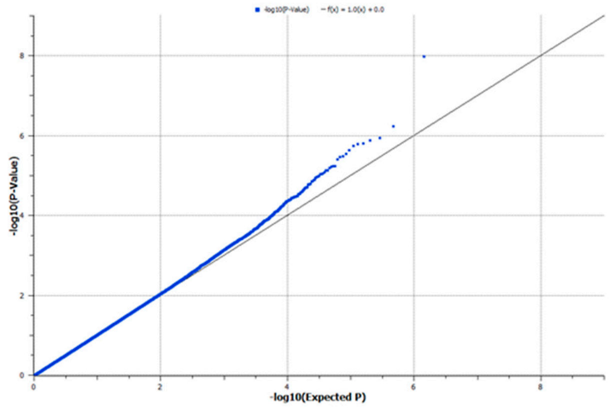

palmitate

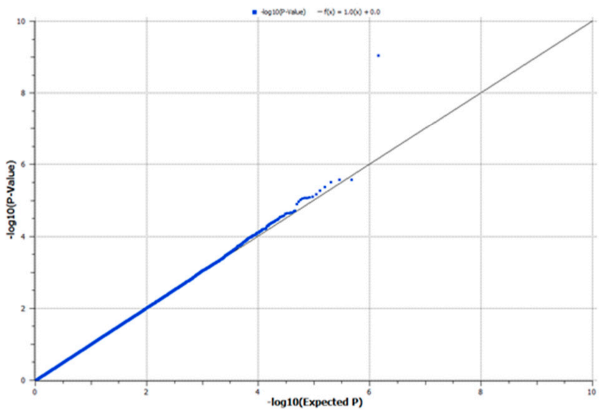

stearate

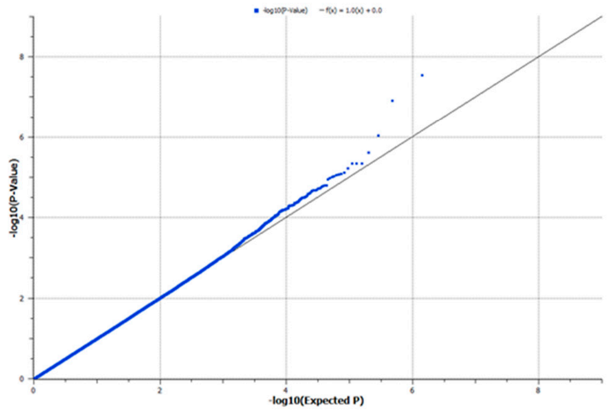

Supplemental Figure 2B:

QQ plots of genome-wide association study of metabolites

10-heptadecenoate

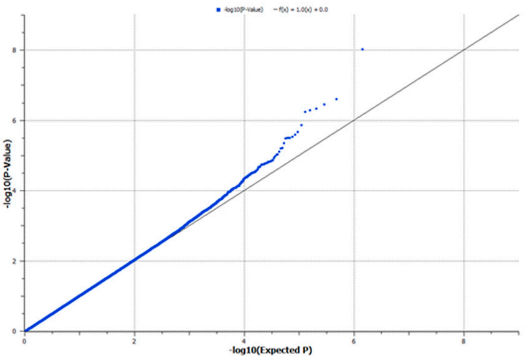

10-nonadecenoate

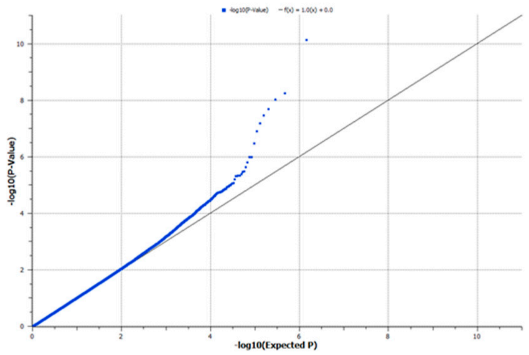

1-palmitoyl-2-arachidonoyl-GPE

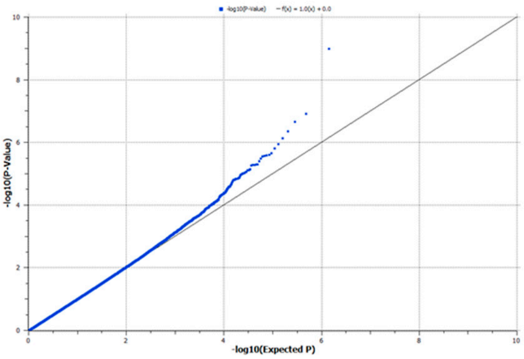

1-palmitoyl-2-lonoleoyl-GPE

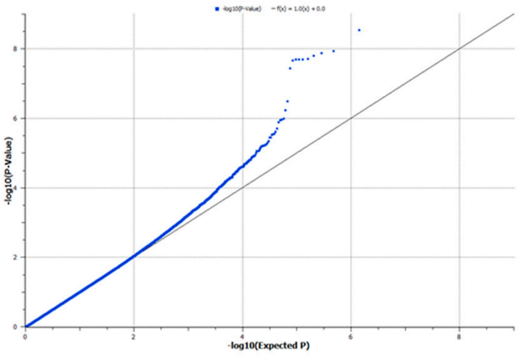

1-palmitoyl-2-oleoyl-GPE

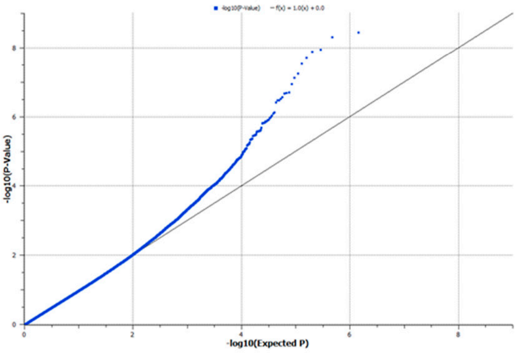

1-stearoyl-2-linoleoyl-GPE

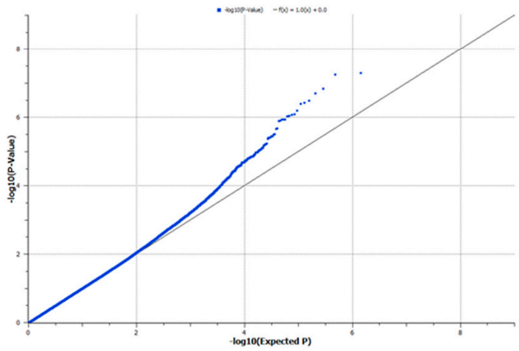

Supplement: Supplementary file 1 [file metabolites-15-00697-s001.zip › Supplemental materials 1.pdf]
